# Accuracy of robot-assisted anterior transpedicular screws in the subaxial cervical spine: an experimental study on human specimens

**DOI:** 10.3389/frobt.2025.1686350

**Published:** 2026-01-07

**Authors:** Lin Cong, Xiaowei Sun, Xiaolu Xi, Ke Yuan, Yajing Cao, Qiang Xie, Yue Zhu

**Affiliations:** 1 Department of Orthopaedics, The First Affiliated Hospital of China Medical University, Shenyang, China; 2 Wuhan United Imaging Surgical Co., Ltd, Wuhan, China; 3 School of Basic Medicine, Tongji Medical College, Huazhong University of Science and Technology, Wuhan, China

**Keywords:** accuracy of screw placement, anterior cervical surgery, anterior transpedicular screw (ATPS), human cadaveric study, robot-assisted surgery

## Abstract

**Study Design:**

Prospective study.

**Objectives:**

This study aimed to evaluate the accuracy and safety of robot-assisted anterior transpedicular screw (ATPS) fixation in human cervical spine specimens.

**Methods:**

A spine robotic system was used to implant thirty-six 1.2 mm Kirschner wires (K-wires) into the cervical pedicles (C4–C7) of five human specimens. Accuracy was assessed by comparing the planned trajectories with the actual K-wire positions. The Gertzbein-Robbins classification system (GRS), adapted for cervical pedicles, was used to evaluate accuracy; Grades A and B (<2 mm pedicle breach) were considered clinically acceptable. Secondary metrics included entry point and angle offsets.

**Results:**

Of the 36 K-wires implanted, nine were placed in C4 and C6, 10 in C5, and eight in C7. According to the adapted GRS, 25 placements (69.4%) were Grade A, 10 (27.8%) were Grade B, and one was Grade C, resulting in a 97.2% clinically acceptable placement rate. The mean target offset was 2.29 ± 1.72 mm, the entry offset was 2.47 ± 1.57 mm, and the angle offset was 5.67° ± 3.72°. No significant differences were observed between the left and right sides (p > 0.05).

**Conclusion:**

Robot-assisted ATPS fixation in cervical specimens achieved high accuracy with 97.2% of placements rated clinically acceptable, indicating its technical feasibility and potential utility in anterior cervical procedures.

## Introduction

To date, studies have shown that patients with severe cervical tricolumnar injuries or those requiring multi-level decompression and reconstruction of the cervical spine may not receive adequate support with a single anterior fixation due to the limited biomechanical stability of the standard anterior cervical screw and plate system (Ye et al., 2024; Bayerl et al., 2019; Sethy et al., 2022). For these patients, supplementary posterior surgery is essential, as screw anchoring can be enhanced by securing the screw in a stronger pedicle (Smith et al., 2016; Singh et al., 2003). However, employing both anterior and posterior surgical methods can lead to prolonged anesthesia, increased trauma, and a heightened risk of neurovascular injury. To address these issues, a surgical technique that offers less trauma, better fixation, and fewer complications is urgently needed in the clinic.

The Cervical Anterior Transpedicular Screw (ATPS), first proposed by Koller et al. (2008), overcomes the conventional anterior approach’s limitations by placing screws into the pedicles to enhance three-column stability, while eliminating the need for posterior surgery (Zhang et al., 2016). Some scholars, including Zhao & Zhu et al., subsequently began applying it in clinical practice (Zhao et al., 2014; Pei et al., 2022). Despite its biomechanical advantages, ATPS remains technically demanding and has not been widely adopted in clinical settings. The presence of vital anterior structures such as the trachea and esophagus, combined with the limited accuracy and stability of current navigation and robotic systems, makes accurate bilateral ATPS insertion in the subaxial cervical spine particularly challenging. As a result, most existing studies have been cadaveric and conducted under fluoroscopic or navigation guidance, with reported accuracy ranging from 76.7% (23/30) to 95% (19/20) (Yukawa et al., 2009; Aramomi et al., 2008). Due to these constraints, only one case report has described robot-assisted ATPS, highlighting the need for systematic validation of its safety and precision (Pei et al., 2022).

At present, X-ray-guided surgical planning is widely used in clinical practice to guide screw implantation (Lin et al., 2022), but this method requires a high level of surgical experience, and its reliability is still concerning. With the clinical application of orthopedic robots, spine surgery has entered the era of intelligence (Zhang et al., 2022). For example, Patton et al. reported that in nine cervical spine specimens with 54 pedicle screws, the accuracy was 42.6% under X-ray fluoroscopy *versus* 66.7% with navigation guidance, a statistically significant difference, demonstrating that navigation technology can improve the learning curve, although current studies indicate that successful positioning (Grade B or better) occurs in only 66.7%–86.1% (Godzik et al., 2019; Bredow et al., 2017; Patton et al., 2015). In the emerging field of robot-assisted spinal surgery, a meta-analysis revealed that, according to the GRS classification, robot-assisted pedicle screw placement achieved significantly higher rates of “perfect” and “clinically acceptable” accuracy compared to conventional freehand techniques (p < 0.05) (Fatima et al., 2021). Widely used robots such as SpineAssist, Renaissance, and Mazor X (Mazor Robotics Ltd., Caesarea, Israel) can reduce human error and soft tissue damage through intraoperative navigation. The accuracy of pedicle localization has improved to between 85.0%–98.2% (Yang et al., 2019; Hyun et al., 2017; Wallace et al., 2020; Vardiman et al., 2020; Zhang et al., 2019; Kim et al., 2017). In the present study, we used a robot-assisted navigation system (uOssa-Nav-s750, China) that belongs to the same category as widely used commercial systems, but with key technical distinctions. The system integrates intraoperative 3D C-arm imaging and point-based rigid registration. It provides semi-automatic trajectory alignment. The robotic arm moves automatically to the preplanned screw trajectory under computer guidance, while the surgeon performs final fine adjustment and locking before drilling. This design ensures surgical safety, allows direct intraoperative control by the surgeon. These features motivated our cadaveric study to quantitatively assess the feasibility and accuracy of robot-assisted ATPS placement and establish reference data for future clinical translation. This study is the first cadaveric experiment to systematically evaluate the safety and accuracy of robot-assisted ATPS placement.

## Materials and methods

Five fresh-frozen adult cadaveric cervical spines (3 males, 2 females; age range 58–75 years; BMI range 19.4–27.8 kg/m^2^) were obtained for this study. All specimens were thawed at room temperature for 24 h before the procedure. Preoperative CT scans were performed to exclude deformities, fractures, or pathological changes. Overall, thirty-six 1.2 mm Kirschner wires (K-wires) were implanted anteriorly into the cervical pedicles (C4-C7). The surgical procedure involved using the spine robotic system (uOssa-Nav-s750, Wuhan United Imaging Surgical Co., Ltd. Wuhan, China). The specimens used in this study were sourced from deceased donors. All specimens were obtained through a formal body donation program with informed consent obtained during the donors’ lifetime for use in scientific research. Thus, this study was exempt from requiring additional informed consent.

### Preoperative design

**FIGURE 1:**
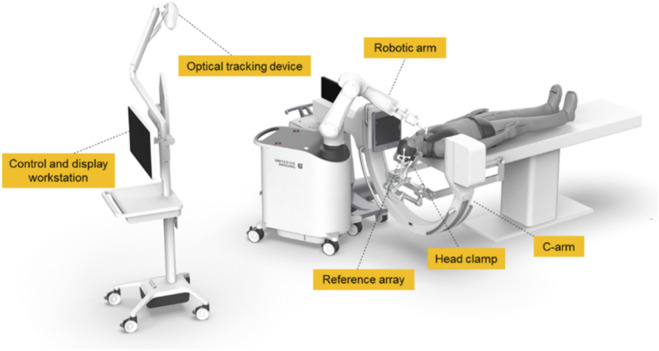
The spine robotic system.

The cadaveric specimen was placed in the supine position on the surgical table, with the head stabilized using a head clamp. The patient reference array was securely attached to the head clamp and positioned adjacent to the surgical field. A PEEK (polyetheretherketone) marker was affixed to the sternum for intraoperative verification of potential specimen displacement and accuracy assessment of the navigation system. Depending on the condition of the specimen, the marker was fixed either with a K-wire. Once secured, the marker remained stable throughout the procedure without interfering with the surgical field. The spine robotic system was positioned on the left side of the specimen. The operating room setup is shown in Figure 1.

**FIGURE 2:**
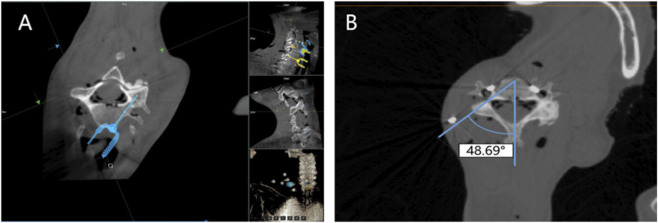
Parameter measurements [**(A)** preoperative planning; **(B)** parameter measurements].

After the specimen was secured, a 3D C-arm (Surgio-B51s, First-imaging, China) was performed for obtaining cervical spine images. Then, the captured images were transferred to the surgical planning software of the spine robotic system for preoperative design. Based on these images, the surgeon planned the trajectory of the anterior cervical pedicle screws on the system monitor. In addition, we measured transverse pedicle height, width and angle by selecting the most appropriate preoperative images using the medical image processing software Mimics (Materialise Leuven, Belgium) (Figure 2).

### Registration and calibration

Before trajectory planning, image-to-patient registration was performed to align the intraoperatively acquired 3D C-arm images with the actual anatomical position of the cadaveric specimen. Five fiducial markers on the patient reference array were identified and matched with their corresponding points in the 3D image data. A rigid point-based registration was then conducted using a singular value decomposition (SVD) algorithm to compute the optimal transformation matrix between the image and patient spaces. The registration accuracy, automatically calculated by the robotic system as the mean target registration error (TRE) across the five paired points, was displayed on the system interface. In all procedures, the mean registration error was consistently below 0.5 mm, indicating high registration precision. During the procedure, the robotic arm was periodically moved toward the sternal marker. This confirmed that no positional drift occurred throughout the surgery. A calibration procedure was carried out using a standard calibration block provided by the manufacturer to verify the spatial correspondence between the drill guide and the robotic coordinate frame.

All procedures were performed by a senior spine surgeon with more than 10 years of experience and over 50 prior robotic-assisted spinal procedures. Each pedicle was drilled once without repeated attempts.

### Surgical procedure

When all the trajectory planning was completed, an anterior surgical approach with a right oblique incision was performed. Subsequently, the surgeon manipulated the robotic system to automatically move the robotic arm close to the planned trajectory, then fine-adjusted and locked. A drill guide tube was inserted into the sleeve holder at the distal end of the robotic arm to create a pilot hole, indicating the planned trajectory. Through the pilot hole, a 1.2 mm K-wire was inserted into the cervical pedicle. With each placement of the K-wire, the robotic arm was moved to the sternal marker to verify that the specimen had not shifted. Additionally, the total time required for preoperative maneuvers, including specimen fixation, reference frame setup, 3D image acquisition, trajectory planning, registration, and calibration, was approximately 17–20 min before the first drilling. This time duration was comparable to that reported for similar spine robotic systems in previous studies.

### Postoperative assessment

After all the K-wire were placed in the pedicle, we performed postoperative CT images (ScintCare CT32, Min Found, China) to assess the accuracy of K-wire placement. We adapted the Gertzbein-Robbins classification system (GRS) for cervical pedicle screws to evaluate the accuracy (Gertzbein and Robbins, 1990). The evaluation involved categorizing into five groups: A, the screw completely contained in the pedicle; B, the cortical penetration of less than 2 mm; C, the cortical penetration of 2 mm or more but less than 4 mm; D, the cortical penetration of 4 mm or more but less than 6 mm; and E, the cortical penetration of 6 mm or more. The grades A and B K-wire positions were considered clinically acceptable, whereas all other grades indicated improper placement.

**FIGURE 3:**
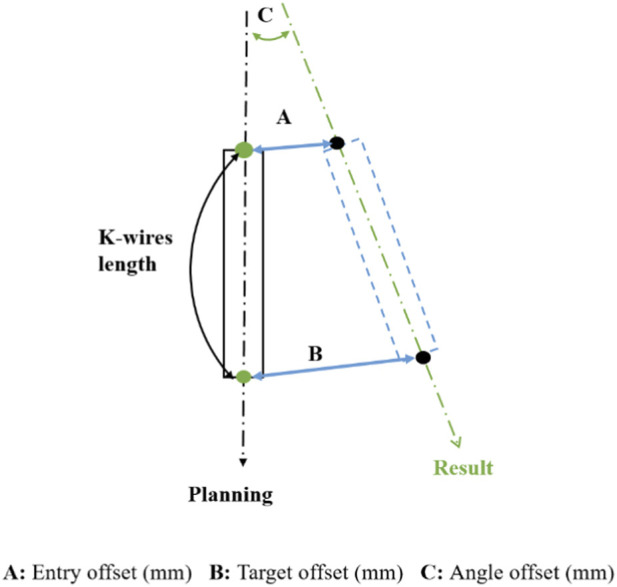
K-wires head, tip, and angle offset accuracy assessment [**(A)** 3-dimensional (3D) distance of placed K-wires head from intended trajectory; **(B)** 3D distance of placed K-wires tip from intended trajectory; **(C)** 3D angular offset of placed K-wires path from planned path].

Furthermore, quantitative measurements were conducted using CT scans to evaluate the accuracy of the K-wire tip, K-wire tail, and K-wire angulation. Postoperative CT images were rigidly aligned with the preoperative plan using the robotic system’s built-in image registration module. The planned and actual K-wire trajectories were visualized and compared within the system software, and quantitative deviations—including tip, tail, and angle offset—were automatically calculated. The accuracy assessment was based on the anterior-most part of the K-wire (tip) and posterior-most part of the K-wire (tail). The angle offset was determined by calculating the difference in the angle between the planned and placed K-wire vectors (Figure 3) (Ryu et al., 2024; Park et al., 2022). For the fairness of the assessment, two senior spine surgeons who were blinded to the treatment group evaluated the accuracy of pedicle screw placement. The consistency test results were substantial (K = 0.740; p < 0.001). When the two evaluation results differed, the final results were determined through a discussion with the first corresponding author of this article.

### Statistical analysis

Descriptive statistics for continuous variables were reported as mean ± standard deviation. Categorical measurements were presented as numbers. The t-test was performed to evaluate the K-wire insertion deviation. All statistical analyses were performed using SPSS 26.0 (IBM, Chicago, IL, United States) and the significance was defined as p < 0.05. Furthermore, a Fisher’s exact test was performed to evaluate the difference in Grade A accuracy between the left and right sides.

## Results

Robot-assisted ATPS fixation was conducted on five freshly frozen cadaveric specimens, and the baseline vertebral physiological parameters of the cadaveric specimens are presented in Table 1.

**TABLE 1:** Preoperative data of the vertebras.

Vertebra	Transverse pedicle height (mm)	Transverse pedicle width (mm)	Transverse pedicle angle (°)
C4	5.45 ± 1.41	4.45 ± 0.23	45.74 ± 1.94
C5	5.54 ± 1.18	5.00 ± 0.28	43.91 ± 7.89
C6	5.87 ± 1.37	5.91 ± 0.07	37.29 ± 3.5
C7	6.65 ± 1.81	7.02 ± 0.13	37.22 ± 5.22

According to the revised GRS (Park et al., 2022), of all the 36 K-wires placements, 25 K-wires placements (69.44%) were grade A. 10 K-wires placements (27.8%) were grade B, and only one placement was Grade C, which occurred on the right side. The majority of left-side K-wires placements (89.47%) were rated as grade A, whereas only 47.06% of right-side placements received this rating (Table 2). Fisher’s exact test indicated that this difference was statistically significant (p = 0.01), suggesting a clear left–right discrepancy in placement accuracy.

**TABLE 2:** Accuracy of screw placement overall.

Grade	Left (n = 19)	Right (n = 17)	Total	/%
A	17 (89.47%)	8 (47.06%)	25	69.44%
B	2 (10.53%)	8 (47.06%)	10	27.78%
C	0	1 (5.88%)	1	2.78%
D	0	0	0	0.00%
E	0	0	0	0.00%

A total of 36 K-wires were implanted. Of these, 9 K-wires were implanted in C4 and C6, 10 K-wires in C5 and 8 K-wires in C7 (Table 3).

**TABLE 3:** Number of screws per vertebra level.

Vertebra	Screw orientation		Total
	Left	Right	
C4	5	4	9
C5	5	5	10
C6	5	4	9
C7	4	4	8

Table 4 illustrates the target deviation between the planning and actual placements of the K-wires when performed in different lateral positions. The deviation of the K-wire placement from the planned trajectory was observed on both the right and left sides, yet these differences were not statistically significant (p > 0.05).

**TABLE 4:** Target deviation from preoperative planning under different lateral positions.

Vertebra	Overall	left (n = 19)	right (n = 17)	P value
	Mean (SD)	Mean (SD)	Mean (SD)	
C4	3.08 ± 2.09	2.89 ± 2.21	3.57 ± 2.48	0.38
C5	2.58 ± 1.53	1.92 ± 1.20	3.41 ± 1.65	0.23
C6	2.43 ± 1.9	2.61 ± 2.27	2.22 ± 1.63	0.47
C7	1.09 ± 0.76	0.77 ± 0.57	1.42 ± 0.86	0.21

Deviation presented as Mean ± SD, in mm.

In the quantitative evaluation of robot-assisted ATPS fixation, the target offset of the actual K-wire position from the planned trajectory was 2.29 ± 1.72 mm. Meanwhile, the entry offset of the actual K-wire position from the planned trajectory was 2.47 ± 1.57 mm, and the angle offset was 5.67° ± 3.72° (Table 5).

**TABLE 5:** Quantitative evaluation of robot-assisted pedicle screw insertion accuracy.

Number of screws	Entry offset (mm)	Target offset (mm)	Angle offset (°)
C4	3.02 ± 1.69	3.08 ± 2.09	7.93 ± 2.93
C5	2.8 ± 1.86	2.58 ± 1.53	6.78 ± 4.15
C6	2.29 ± 1.41	2.43 ± 1.9	5.18 ± 3.67
C7	1.81 ± 1.29	1.09 ± 0.76	2.98 ± 2.36
Total	2.47 ± 1.57	2.29 ± 1.72	5.67 ± 3.72

Deviation presented as Mean ± SD, in mm.

**FIGURE 4:**
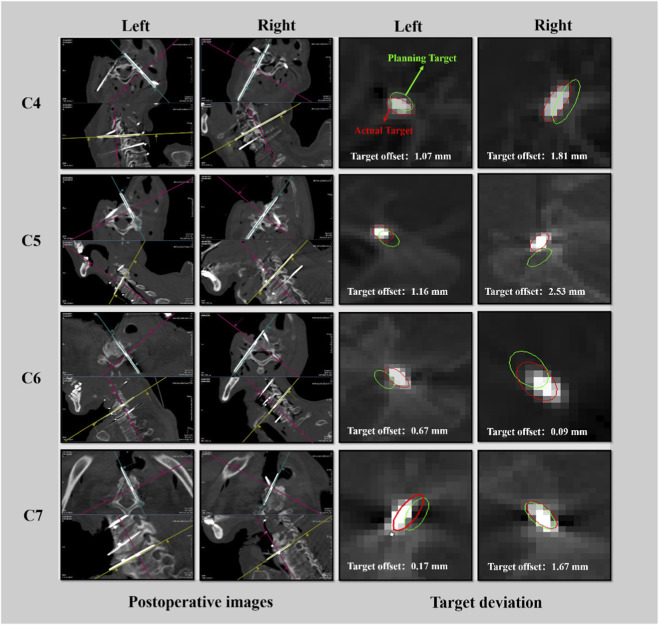
Postoperative evaluation of the C4-C7 spine.

Among these, Figure 4 demonstrates the postoperative imaging results of the C4-C7 spinal segment in one of the cadaver specimens.

## Discussion

This study evaluated the accuracy and safety of robot-assisted ATPS in human specimens. A total of 36 K-wires were successfully placed under the guidance of the robot on the left and right sides of the C4-C7 cervical vertebrae in five cadaveric specimens, and no failure was found. Despite our grade A accuracy of 69.44%, the “clinically acceptable” (or G&R grade A+ B) implantation accuracy for all K-wires implanted was 97.22%, significantly superior to those reported in previous studies on the accuracy of anterior cervical pedicle placement applying navigation (Bredow et al., 2017; Patton et al., 2015).

Compared with traditional anterior and posterior surgery, ATPS fixation combines the benefits of both anterior and posterior cervical surgery, allowing treatment of cervical spondylosis with anterior surgery while also providing the stability of pedicle screw fixation (Pei et al., 2022). As a novel supplement to the traditional cervical pedicle screw approach, ATPS has been shown to offer greater stability for multi-segment and severe tricolumnar injuries, with some success in clinical applications (Ye et al., 2024; Koller et al., 2008; Zhang et al., 2022). Conventional screw placement is usually performed under fluoroscopy, and the need to continuously acquire images during the procedure not only makes the entire process very tedious, but more importantly, exposes the patient and the surgeon to prolonged radiation due to repeated fluoroscopy to adjust or confirm the screw placement. In particular, placement of ATPS is more difficult than either anterior vertebral body screws or posterior transpedicular fixation due to few landmarks on the anterior surface of cervical vertebrae and a relatively long distance between the anterior surface and the pedicle (Koller et al., 2008). The advent of robot-assisted surgery greatly mitigate this issue, and it provides a high level of accuracy. The surgical robot has a sensitive optical tracking system and a flexible robotic arm that works together, even if the actual position of the patient changes during the operation, the reliability and repeatability of the planned screw path and direction can be ensured by accurate positioning on the preplanned pedicle screw trajectory (Li et al., 2020). At present, there have been numerous reports on robot-assisted spinal screw placement (Lin et al., 2022; Godzik et al., 2019). Nevertheless, for this unconventional ATPS operation, there is only one case report on robot-assisted ATPS combined with 3D-printed implants in the treatment of multiple cervical spine fractures, published in 2022, which demonstrated the potential of robot-assisted ATPS in clinical application (Pei et al., 2022). The robotic system in this study, through accurate planning and precise manipulation, increased the precision of screw placement to 97.2%, thereby substantiating the assertion that this intricate procedure can be executed with greater accuracy and security through the assistance of a robotic system.

Compared with previous 2022 clinical case report, which described the feasibility of robot-assisted ATPS combined with 3D-printed implants in a single patient, the present cadaveric study provides a systematic and quantitative evaluation of screw placement accuracy under controlled experimental conditions. The current study measured objective parameters such as entry/target offsets and angular deviations, thereby validating the precision and safety of the robotic system beyond a single clinical demonstration. These findings extend the earlier feasibility evidence into a reproducible quantitative framework, forming the experimental basis for future clinical trials.

The GRS was found to be an effective clinical evaluation system in our study. However, it still has some shortcomings in evaluating the accuracy of spinal surgery with robot-assisted screw placement. In previous studies, Ryu et al. (2024) simply established the standard of acceptable deviation ranges based on the results reported by himself and Godzik et al. (Zhang et al., 2016): an entry offset and target offset of less than 2 mm, and an angle offset of less than 5°. According to Godzik et al., a total of 70 screws were placed in 17 patients, with an average 2D accuracy of 2.6 ± 1.1 mm and an angular deviation of 5.6° ± 4.3°. Among the 36 K-wires placed in this study, the total target deviation was 2.29 ± 1.72 mm and the total angular deviation was 5.67° ± 3.72°, which were similar to the results of aforementioned study. Although our study primarily evaluated the placement accuracy of robot-assisted ATPS, it did not directly measure reproducibility because each trajectory was executed once per specimen. Nevertheless, the robotic platform integrates automatic trajectory positioning and optical navigation, which largely eliminates operator-dependent variability in hand–eye coordination and instrument handling. The relatively small dispersion in the measured deviations across vertebral levels indirectly reflects a high degree of procedural consistency. Future studies involving repeated insertions by different operators and under varied conditions are warranted to quantitatively confirm the reproducibility advantage of the robotic system.

Although the overall accuracy achieved in this study was high (97.2% clinically acceptable (Grade A+ B), 95% CI: 85.5%–99.9%), the results should be interpreted with caution due to the clustered nature of the data, as multiple trajectories were derived from the same specimen. The statistical analysis using Fisher’s exact test revealed a significant difference in Grade A accuracy between the left and right sides (89.5% vs. 47.1%, p = 0.01), and the only instance of failure was observed in a case on the right side of C4This discrepancy is most likely attributable to the surgical approach and operative ergonomics rather than intrinsic robotic errors. In addition, fresh frozen specimens undergo tissue changes, resulting in less feedback when the drill makes contact with cortical bone (Bredow et al., 2017).

All procedures were performed through a right-sided anterior cervical approach, with the surgeon standing on the patient’s left side. This provided a wider view and easier manipulation for the left pedicle trajectories, while the right side required a steeper insertion angle and deeper retraction, reducing operational freedom and visualization. Importantly, the robotic system itself was not a major source of asymmetry: the robotic arm’s range of motion was sufficient throughout all procedures, and intraoperative verification confirmed that registration accuracy remained within 0.5 mm. These findings suggest that the geometric constraints of the right-sided approach, especially retractor interference and soft-tissue obstruction, likely accounted for the slightly higher deviation on the right side.

Future optimization of approach geometry, retractor design, and workspace planning may help achieve more symmetric bilateral accuracy. Moreover, given the limited sample size and potential intra-specimen correlation, future studies should employ larger datasets and advanced statistical models to confirm these findings and quantify the impact of surgical ergonomics on robot-assisted ATPS performance.

In this experiment, we also encountered certain challenges in K-wire placement for the C4 and C7 segments. Although retractors were used to expand exposure, the anatomical positions of C4 and C7 lie at the upper and lower ends of the incision, limiting the field of exposure and requiring steeper placement angles. Therefore, to facilitate K-wire placement into the C4 segment, the surgical incision could be modified to adopt the Macfee surgical approach, but this would increase the surgical trauma area. For the C5 and C6 segments, the surgical incisions provided clear exposure under traction, facilitating the surgical procedure. In addition, many current articles only use local specimens (frozen, neck) when performing ATPS surgery, and do not consider the influence of specimen pulling on the screw placement during surgery. As the whole cadaver specimens was used in our study, the fixation of specimens should be considered throughout the operation. For specimens with low body weight, the lack of whole body fixation during pulling process will result in overall specimen displacement, which may affect the accuracy of K-wire placement.

We believe that further research on ATPS is highly valuable due to its numerous theoretical advantages. The anterior approach alone should significantly reduce morbidity and shorten operative time. Additionally, robot-assisted technology can help surgeons better plan and simulate pedicle screw placement, thereby enhancing accuracy and the success rate of pedicle screw placement. Precisely positioning the placement points can minimize risks by avoiding critical areas, reducing damage to the vertebral joint, pedicle, and surrounding soft tissues, and thereby decreasing surgical risks and blood loss. However, our study also has certain limitations. First, the current study used only a small number of cadaver specimens, and a larger sample study is needed to obtain more convincing results. Second, cadaver studies cannot account for intraoperative bleeding or simulate tissue movement due to the patient’s breathing. Third, since cadaver experiments do not consider the risk of esophageal and tracheal injury caused by retractors in clinical applications, these risks may be present in clinical practice. In the future, we will conduct further clinical trials to verify the precision and safety of robot-assisted ATPS surgery.

## Conclusion

This study validated the safety and accuracy of a robotic system for anterior cervical transpedicular screw fixation. ATPS provides both the stability of posterior fixation and the minimally invasive advantages of anterior surgery, making it particularly beneficial for elderly patients with osteoporosis or multilevel cervical injuries. However, its clinical adoption remains limited due to the high technical difficulty and risk of neurovascular injury. The proposed robotic system enables precise execution of screw trajectories. With a 97.2% clinically acceptable accuracy rate, the results demonstrate that robot-assisted ATPS fixation is a feasible and safe approach. This system represents an important step toward intelligent and data-driven cervical spine surgery, enhancing the reproducibility and safety of complex anterior spinal procedures.

## Article summary

### Article focus

The anterior transpedicular screw (ATPS) fixation procedure represents a novel surgical technique applied in the treatment of a diverse array of cervical spine disorders, but there are few studies on robot-assisted ATPS fixation.

The aim of the current study was to evaluate the accuracy and security of robot-assisted ATPS fixation on human specimens.

### Key messages

The accurate positioning rate of K-wire placement was 97.2% (grade B or higher) in robot-assisted ATPS.

Robot-assisted anterior cervical pedicle screw placement is a feasible approach, providing an alternative to traditional anterior cervical surgery.

### Strengths and limitations of this study

The accuracy and security of robot-assisted ATPS was verified by an experiment in which 36 K-wires were implanted anteriorly into the cervical pedicle (C4-C7) of five human specimens.

The current study utilised only a small number of cadaver specimens and cadaver study cannot account for intraoperative bleeding or simulate tissue movement due to the patient’s breathing.

## Data Availability

The original contributions presented in the study are included in the article/supplementary material, further inquiries can be directed to the corresponding author.
